# Emergence of Delayed Methylmercury Toxicity after Perinatal Exposure in Metallothionein-Null and Wild-Type C57BL Mice

**DOI:** 10.1289/ehp.10906

**Published:** 2008-02-26

**Authors:** Minoru Yoshida, Natsuki Shimizu, Megumi Suzuki, Chiho Watanabe, Masahiko Satoh, Kouki Mori, Akira Yasutake

**Affiliations:** 1 Faculty of Human Health Sciences, Hachinohe University, Hachinohe, Japan; 2 Department of Chemistry, Meisei University, Hino, Tokyo, Japan; 3 Department of Human Ecology, Graduate School of Medicine, University of Tokyo, Tokyo, Japan; 4 Laboratory of Pharmaceutical Health Sciences, School of Pharmacy, Aichi Gakuin University, Nagoya, Japan; 5 Division of Nephrology, Endocrinology and Vascular Medicine, Tohoku University Graduate School of Medicine, Sendai, Japan; 6 Biochemistry Section, National Institute for Minamata Disease, Minamata, Kumamoto, Japan

**Keywords:** behavioral effects, latency, methylmercury, mice, perinatal exposure

## Abstract

**Background:**

Although a long latency period of toxicity after exposure to methylmercury (MeHg) is known to exist in humans, few animal studies have addressed this issue. Substantiation of delayed MeHg toxicity in animals would affect the risk evaluation of MeHg.

**Objectives:**

Our goal in this study was to demonstrate the existence of a latency period in a rodent model in which the toxicity of perinatal MeHg exposure becomes apparent only later in life. Our study included metallothionein (MT) knockout mice because studies have suggested the potential susceptibility of this strain to the neurodevelopmental toxicity of MeHg.

**Methods:**

Pregnant MT-null and wild-type C57Bl/6J mice were exposed to MeHg through their diet containing 5 μg Hg/g during gestation and early lactation. We examined behavioral functions of the offspring using frequently used paradigms, including open field behavior (OPF), passive avoidance (PA), and the Morris water maze (MM), at ages of 12–13 and 52–53 weeks.

**Results:**

At 12 weeks of age, behavioral effects of MeHg were not detected, except for OPF performance in MeHg-exposed MT-null females. At 52 weeks of age, the MeHg-exposed groups showed poorer performance both in PA and MM, and their OPF activity differed from controls. These effects of MeHg appeared exaggerated in the MT-null strain. The brain Hg concentration had leveled off by 13 weeks of age.

**Conclusions:**

The results suggest the existence of a long latency period after perinatal exposure to low-level MeHg, in which the behavioral effects emerged long after the leveling-off of brain Hg levels. Hence, the initial toxicologic event responsible for the late effects should have occurred before this leveling-off of brain Hg.

Methylmercury (MeHg) poses serious and practical concerns for human populations regarding perinatal exposure. Fish, especially large predator (carnivore) fish species, accumulate high concentrations of MeHg through the marine food chain, and exposure of pregnant women to MeHg through the consumption of fish has evoked widespread concern due to potential effects on offspring. Two large-scale cohort studies in fish-eating populations of Seychelles and Faroe islanders are being conducted; although the former has not found consistent adverse developmental effects of MeHg (Myers et al. 2007), the latter has reported adverse effects (Debes et al. 2006). Fundamental reasons for this discrepancy have not been completely elucidated, and many questions remain regarding the neurotoxicity of MeHg, despite extensive study.

Among the unanswered questions is whether there is a long latency period for behavioral manifestations after exposure to MeHg (Clarkson and Magos 2006; Landrigan et al. 2005; Rice 1996; Weiss et al. 2005a, 2005b). Typical examples of latent toxicity in humans, including both acute and chronic MeHg exposures, have been described in detail elsewhere (Weiss et al. 2005a). Davidson et al. (2006) recently suggested that effects of perinatal exposure to MeHg may emerge 9 years after birth in the Seychelles cohort. Consequently, risk assessments of MeHg exposure could be inaccurate because studies (human or animal) usually do not focus on later stages of life and therefore could miss delayed effects. The possibility of delayed toxicity is exemplified by the expanded Barker hypothesis, which posits that the origin of some neurodegenerative diseases such as Parkinson and Alzheimer diseases lies in early exposure to environmental chemicals (Landrigan et al. 2005). Although epidemiologic evidence would be ideal for exploring the possibility of delayed toxicity (and, indeed, data from epidemiologic studies form the basis of current risk assessment for developmental toxicity of MeHg), considering the complex effects of numerous potential confounders and the existence of multiple exposures in human populations, animal models would likely make important contributions to this field.

Although numerous animal studies have described the developmental neurotoxicity of MeHg (Watanabe and Satoh, 1996), few have addressed the latency issue. Few studies have evaluated the neurobehavioral effects in rodents longitudinally beyond 6 months after perinatal exposure. Spyker (1975) addressed this issue in her pioneering work, reporting the late development of behavioral toxicity in mice prenatally exposed to MeHg; it appeared, however, that the substantial mortality and retarded growth among the exposed mice were apparent before weaning, indicating that the doses used (even though some lower dose levels were included) exerted severe toxicity. Using a relatively complex schedule-controlled operant behavior method, rats whose parents were exposed to MeHg (0.5 or 6.4 mg/L) from 4 weeks before mating and continuing to postnatal day (PND) 16 were shown to be less sensitive to a change in the reinforcement schedule than were their nonexposed counterparts at 28–32 months of age (Newland et al. 2004). Mice that were perinatally exposed to 1 or 3 mg/L MeHg in drinking water did not show significant deviation from controls in behavioral performance (motor performance, memory, and learning) at 5, 15, or 26 months of age, but the lifetime-exposed groups did show a significant deviation (Weiss et al. 2005c). The existence of a latency period (i.e., the absence of effects earlier in life followed by the emergence of effects at a later stage of life) has not been demonstrated in any rodent study. In nonhuman primates, delayed emergence of the signs of neurotoxicity was observed several years after the cessation of a 7-year postnatal exposure (Rice 1996).

Metallothionein (MT) protects against the toxicities of a variety of metals. We examined the neurotoxicity and developmental toxicity of metallic Hg in MT I/II-knockout mice (Yoshida et al. 2004) and demonstrated the susceptibility of this genetically manipulated strain to the toxicity of metallic Hg. In contrast to metallic Hg, MeHg does not induce MT, and MT would not substantially influence the kinetics of MeHg (Yasutake et al. 1998). Several reports, however, have demonstrated protective effects of MT against MeHg toxicity, which was ascribed to the radical scavenging effect of MT (Yao et al. 2000). We also showed that perinatal exposure to MeHg results in altered metabolism of thyroid hormones in neonates that was more distinct in MT-null strains than their wild-type counterparts (Mori et al. 2006). The vulnerability of the MT-null strain suggests that delayed neurobehavioral toxicity due to MeHg, if it does exist, might be more distinctive in this strain.

By utilizing the MT-null strain, we aimed to answer the following two questions: First, could we generate a model in which the toxicity of MeHg would emerge or at least become exaggerated later in life as opposed to earlier in life (i.e., at 3–6 months, which was the timing for most of the earlier studies that used behavioral evaluations)? Second, would the MT-null strain be affected more than its parent C57BL strain? Answering either of these questions not only could influence the risk evaluation of MeHg, but it could also lead to a better understanding of the mechanism of toxicity for perinatal MeHg exposure. To address these issues, we used three behavioral paradigms, the open field (OPF), passive avoidance (PA), and Morris (water) maze (MM) tests, which are often used in this field and which we used in our previous studies on the effects of Hg vapor (Yoshida et al. 2004, 2006). Performances in the MM and PA are said to be the most sensitive to aging (Gower and Lamberty 1993).

We used a dose of 5 μg MeHg/g in the diet, which resulted in a brain Hg level relevant to human risk assessment. We evaluated the behavioral end points twice, once around 3 months of age and the other time around 1 year; the latter time roughly corresponds to the period when many behavioral performances, including OPF (Acevedoa et al. 2006; Carrie et al. 1999; Gower and Lamberty 1993), PA (Gower and Lamberty 1993), and MM (Bach et al. 1999; Carrie et al. 1999), show alterations in this mouse strain.

## Materials and Methods

### Animals and MeHg exposure

OLA129/C57BL/6J strain mice (wild type) and MT I/II-knockout mice (MT-null) of this strain were provided by K.H. Choo of the Murdoch Institute, Parkville, Australia (Michalska and Choo 1993) and were of a mixed genetic background of 129/Ola and C57BL/6 strains. F_1_ hybrid mice were mated with C57BL/6 mice for six generations at the National Institute for Environmental Studies (Tsukuba, Japan). At 10 weeks of age, single male and female mice were allowed to cohabit; every female mouse was checked each morning for the presence of a vaginal plug. When a plug was confirmed, the day was designated as day 0 of gestation (GD0).

The diet, NIH-07PLD formula (CLEA Japan, Inc., Tokyo, Japan), contained vitamins and trace elements as follows (per kilogram diet): 3.2 mg CuSO_4_, 88 mg FeSO_4_, 149 mg MnSO_4_, 25 mg ZnCO_3_, 1.6 mg Ca(IO_3_)_2_, 11 mg vitamin B_1_, 4.7 mg vitamin B_2_, 1.9 mg vitamin B_6_, 44 mg vitamin E, in addition to 5 μg MeHg/g. This diet was fed to the pregnant mice starting from GD0 through 10 days after delivery (i.e., PND10). Thereafter, we switched mice to a diet that did not contain MeHg. We chose GD0 as the beginning of exposure because exposures that started before conception often resulted in fairly high Hg concentrations in fetal/neonatal brains (Kakita et al. 2003), and we chose PND10 to cover the early neonatal period, in which considerable brain growth occurs. In our experimental setting, the neonatal mice started to eat from the diet bucket and drink from the water bottle from PND10 onward. Control mice were kept on the same diet but without MeHg (< 0.01 μg Hg/g). On PND1, to avoid the confounding effects due to different litter size, we reduced each litter to six pups (three males and three females when possible), and on PND10, two males and two females from each litter were killed for chemical analyses.

The remaining male and female offspring per litter were weaned on PND28 and used for subsequent behavioral analyses (either at 12–13 weeks or 52–53 weeks, depending on the litter) as described below. We measured body weights of the weaned mice every 2 weeks. Thus, four experimental groups were used (with or without MeHg exposure for two strains), and each group consisted of 12–13 litters. For half of the litters, the behavioral analyses were conducted at 12–13 weeks of age, and upon completion of the behavioral analyses, the animals were killed under ether anesthesia. We then dissected the organs (brain, liver, and kidneys) for Hg analyses. For the remaining half of the litters, we conducted the behavioral tests at 52–53 weeks of age. The mice were treated humanely and with regard to alleviation of suffering according to the National Institute for Environmental Studies’ Guidelines for Animal Welfare and the guidelines of St. Marianna University.

### Behavioral evaluations

The details of each behavioral procedure have been described elsewhere (Yoshida et al. 2006). Brief descriptions follow.

For the OPF test, we used an OPF apparatus (Ohara Co., Ltd., Tokyo, Japan) with a 60 × 60-cm floor surrounded by walls 60 cm high. The experimental room light was turned off, and a dim light of 80 lux was lit during the experiment. We placed a mouse in the center of the floor and monitored its behavior for 10 min using a CCD camera connected to a computer. The position of the center of gravity was calculated by image-analyzing software, which was used to calculate the total distance traveled by the mouse as well as the positional preference (either center or peripheral, where peripheral was defined as the area within 10 cm from the wall). We cleaned the OPF apparatus with 70% ethanol between trials.

The apparatus for the PA test (Ohara Co. Ltd.) consisted of a light compartment that illuminated by a 400-lux light and a dark compartment with black opaque walls and lids. The two compartments were separated by a mobile guillotine door. On the first day (training trial), we placed each mouse in the light compartment facing away from the guillotine door, which was closed. After 30 sec of introduction, the door was opened; when the mouse entered the dark compartment, a brief electric shock (4 mA for 2 sec) was delivered through the metal grid floor. This would force the mouse back to the light component. The interval between the opening of the door and the entry to the dark room (in seconds) was recorded and defined as the latency. On the next day, the same procedure was repeated, but without the electric shock (retaining trail). In this PA paradigm, aversive learning was assumed to be established in the training trial, and we used its retention in the retaining trail as the index of learning. Between each individual trial, we cleaned the apparatus with ethanol. The cutoff time of the retention session was 300 sec.

The MM test apparatus (Ohara Co., Ltd.) was a round-shaped water pool with a diameter of 120 cm. A small platform was submerged in the water, which provided a place for mice to escape from the water (i.e., an aversive stimulus, water temperature = 23 ± 1°C). The water was made opaque by adding white paint so that the mouse could not see the submerged platform. In each trial, we released a mouse into the pool from a determined position along the wall, and the performance of the mouse was monitored by a CCD camera/image analyzer. The time required to reach the platform was recorded. If a mouse could not find the platform within 60 sec after release, it was led to the platform and placed on it for 20 sec before being removed. In these cases, a latency of 60 sec was recorded. We conducted the trial once a day up to the fifth day for each mouse, and the order of each mouse was counterbalanced across the day. On the sixth day, a transfer test (or probe test), which is a trial without the platform, was conducted; in this procedure, we counted the number of times that the mouse crossed the position where the platform had been.

### Tissue Hg concentration

The tissue samples were homogenized (10% weight/volume) in distilled water using a Polytron homogenizer (Kinematica GmbH, Littau, Switzerland). We determined Hg levels in the homogenates by the oxygen combustion–gold amalgamation method (Ohkawa et al. 1977) using an atomic absorption Hg detector MD-1 (Nippon Instruments, Co. Ltd., Osaka, Japan). To ensure the accuracy of the measurement, we included reference material from a dogfish (DORM-2; National Research Council of Canada, Ottawa, Ontario, Canada) with a certified value of 4.64 ± 0.26 μg/g in the analyses; the observed values fell within the certified range. The detection limit of the measurement was 0.1 ng Hg.

### Statistics

We analyzed behavioral data for OPF and PA by analysis of variance (ANOVA), taking sex, strain, and MeHg exposure as the factors. All the interactions among these factors were put into the model. When any of the interactions was highly significant, we analyzed the data separately in an appropriate way; for example, if sex × Hg was significant, the data for males and females were separately analyzed for the effects of strain and Hg. Whenever appropriate, ANOVAs were followed by Mann-Whitney *U* or Student’s *t*-tests, depending on the nature and distribution of the variables. We analyzed data for the MM by repeated-measures ANOVA, taking the exposure as between-group and trials as within-group variables. The test was performed for each of the four sex and strain combinations separately. The significance level was set at *p* < 0.05.

## Results

### Body weight

**Figure 1:**
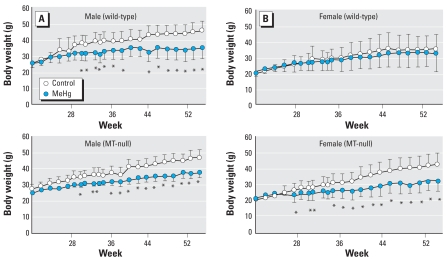
The change in body weight (mean ± SD) of wild-type and MT-null male (*A*) and female (*B*) offspring perinatally exposed to MeHg over time; *n* = 6–7 mice per group. *Significant difference between the MeHg-exposed mice and their corresponding controls.

Up to 20 weeks of age, the body weight values of the control and MeHg-exposed groups were not different, regardless of strain or sex. After 28 weeks, except for the wild-type female groups, the MeHg-exposed groups weighed significantly less than the controls (Figure 1).

### OPF

**Figure 2:**
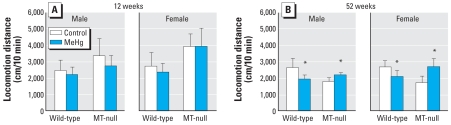
Effect of MeHg exposure on locomotion distance (mean ± SD) during the 10-min OPF session at 12 weeks (*A*) and 52 weeks (*B*) of age. At 52 weeks (*B*), exposure to MeHg exerted significant effects, with decreases in locomotion in the wild-type but increases in the MT-null mice, leading to the significant strain × Hg interaction (*n* = 6–7 mice per group for each time point). *Significant difference between the MeHg-exposed mice and their corresponding controls.

**Figure 3:**
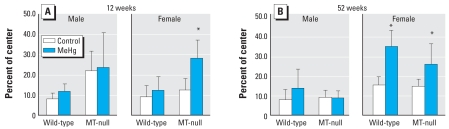
Effect of MeHg exposure on proportion of time occupying any of the center areas (9 areas of 25, which were not touched with the walls) (mean ± SD) in wild-type and MT-null male and female mice during the 10-min session at 12 weeks (*A*) and 52 weeks (*B*) of age; *n* = 6–7 mice per group for each time point. *Significant difference between the MeHg-exposed mice and their corresponding controls.

At 12 weeks of age, a three-way ANOVA of the locomotion distance revealed that only strain was a significant factor (*p* < 0.001), reflecting the longer distance traveled by the MT-null mice (Figure 2A). Strain was also a significant factor for the proportion of the central-area locomotion (Figure 3A), and Hg exposure marginally affected this outcome. In MT-null females, the proportion of central-area locomotion was higher in MeHg-exposed mice than in the controls; this difference was not observed in any other strain–sex combination. At 52 weeks of age, the strain × Hg interaction was highly significant (*p* < 0.001) in an ANOVA of locomotion distance (Figure 2B); MeHg exposure was associated with decreased locomotion distance in wild-type mice and with increased distance in MT-null mice. A strain-wise two-way ANOVA (with sex and Hg as the factors) revealed that only Hg was significant in both strains (*p* < 0.01). Regarding the proportion of the central-area locomotion, the effects of MeHg appeared to depend on sex [i.e., sex × Hg was highly significant (*p* < 0.001) in the three-way ANOVA; Figure 3B]. Indeed, a sex-wise two-way ANOVA showed significant effects of Hg only in females (*p* < 0.001).

### PA

**Figure 4:**
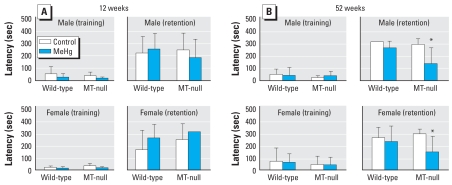
Effect of MeHg exposure on escape latency from the illuminated to the dark compartment in the PA test (mean ± SD) at 12 weeks (*A*) and 52 weeks (*B*) of age; *n* = 6–7 per group for each time point. *Significant difference between the MeHg-exposed mice and their corresponding controls.

At 12 weeks of age, all the groups showed prolonged latency in the second (retention) trial, and no consistent effect of MeHg was recognized regardless of strain or sex (Figure 4A).

At 52 weeks of age, a three-way ANOVA revealed a significant interaction between strain and Hg (*p* < 0.05); strain-wise two-way ANOVAs revealed a significant effect of Hg on learning in MT-null mice of both sexes; these groups of mice showed significantly shorter (less than half) latency times compared to control mice (Figure 4B). A notable difference between the results at 52 weeks of age and those at 12 weeks was that many of the tested mice exceeded the cutoff time in the retention trials at 52 weeks, except for the MT-null groups.

### MM

**Figure 5:**
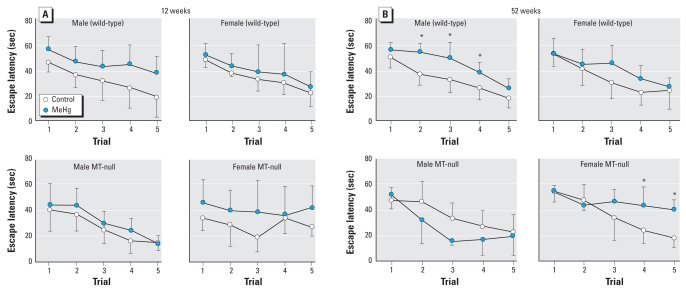
The escape latency in the MM (mean ± SD) at 12 weeks (*A*) and 52 weeks (*B*) of age; *n* = 6–7 per group for each time point. *Significant difference between the MeHg-exposed mice and their corresponding controls.

At 13 weeks of age, repeated-measures ANOVA did not indicate any effects of MeHg (Figure 5A). At 52 weeks of age (Figure 5B), wild-type males and MT-null females shared the same statistical results; Hg as well as the Hg × trial interaction were statistically significant. Thus, in both cases, the MeHg groups showed a longer latency, hampering learning performance.

### Tissue Hg concentration

At PND10, which was immediately after the exposure, brain Hg concentrations of the neonatal mice were approximately ≤ 0.5 μg/g (Table 1). Although the MT-null mice and females showed slightly higher brain Hg concentrations than the corresponding wild-type group and males, respectively, neither of these differences was significant. At 13 weeks, when the behavioral tests were completed, the brain Hg concentration was comparable to the control (nonexposed) level (approximately 5 ng/g in both the exposed and control brains). Interestingly, MT-null mice had a significantly lower brain Hg concentration than the corresponding wild-type groups.

## Discussion

Results of the present study demonstrate the delayed emergence of neurobehavioral toxicity due to perinatal MeHg exposure, which presumably developed after brain MeHg concentrations had leveled off. This emergent toxicity was exaggerated in MT-null mice and was more distinct in females. To our knowledge, our findings show the first clear-cut demonstrations of a long latency period of MeHg neurobehavioral toxicity in rodents and possible genetic susceptibility for the emergent toxicity.

The exposure level should be considered before discussing the end points. On PND10, immediately after the cessation of MeHg exposure, the brain Hg concentration was approximately 0.5 μg/g, regardless of the strain or sex. In a previous study, the brain Hg concentration in mice perinatally exposed to 6 mg MeHg/L (via water) peaked between PND0 and PND4 and was approximately three times higher than on PND21 (Goulet et al. 2003). Therefore, the peak brain Hg concentration, which is presumably observed around birth, can be estimated as about 3-fold higher than that on PND10 and would be approximately 1.5 μg/g (0.5 μg/g × 3), which is one of the lowest levels among recent rodent studies. As shown by Sakamoto et al. (2002) in their Figure 2, prenatal exposure of rats to MeHg showed a peak brain Hg concentration around PND1 that was four to five times greater than the level on PND10. Also, Newland and Rasmussen (2000) reported a slight alteration of a complex operant behavior in rats at ages < 2 years at brain Hg concentrations as low as 0.5 μg/g at birth, although statistical significance of this particular effect was not clear. It should be noted that rats have different Hg kinetics (Hirayama and Yasutake 1986; Yasutake and Hirayama 1990) due to the high affinity of rat hemoglobin for MeHg (Clarkson and Magos 2006).

The most important observation of the present study was that the effects of low-level MeHg exposure were detected only at later stages in the lives of the mice. Except for the central-area occupancy in OPF in MT-null females, no statistically significant effects of MeHg were observed in any of the three behavioral tests at 12 weeks of age. In contrast, significant effects were observed in all three tests at 52 weeks of age. The brain Hg concentration of the exposed groups had leveled off and was not distinguishable from the non-exposed group at 13 weeks of age, immediately after the first phase of the behavioral testing. Therefore, in the present study, there was a latency period in which the dose and effects could not be detected, although effects were observed 9 months later. Another notable observation was that the emergent manifestation of toxicity was also recognized in the suppression of body weight (except for wild-type females), which only became apparent on or after 28 weeks of age.

The existence of a latency period of as long as several years after chronic (7 years from birth), low-level exposure to MeHg has been described in nonhuman primates (Rice 1996). In that case, however, the Hg concentration in the brain remained elevated, presumably as a result of the long exposure. Indeed, Rice (1996) argued that the minute amount of residual brain Hg could have caused the delayed toxicity. This was clearly not the case in the present study because the Hg concentration leveled off around the time of the first phase of the behavioral study. The absence of behavioral effects at 12 weeks of age ruled out the possibility that the residual behavioral effects were due to elevated Hg early in life. Therefore, the behavioral toxicity that surfaced at 52 weeks of age must have had its origin before the brain Hg concentration leveled off (at or before 13 weeks of age), although the redistribution of Hg to the brain from other sites of deposition, such as the liver, cannot be completely excluded. The long silent period before the manifestations of toxicity emerged suggests that a slow process plays a role in this latent toxicity.

Although an example of a slow process is aging, 52 weeks of age might not be sufficiently old for a mouse to be considered aged in the physiologic sense because C57BL/6 mice have a relatively long life span among mouse strains [median survival of 27–31 months (Gower and Lamberty 1993)] and a survival rate at 18–19 months as high as 90% (Institute on Aging HP) (National Institute on Aging 2008). Nevertheless, various behavioral examinations have shown age-related changes in the performance of mice at approximately 1 year of age in the OPF (Acevedoa et al. 2006; Carrie et al. 1999), MM (Bach et al. 1999; Carrie et al. 1999), and PA (Gower and Lamberty 1993) tests. The observed effects of MeHg, including the deterioration in the PA and MM and suppression in the OPF (in wild-type mice), were consistent with these reported effects of aging on behavioral function (in the sense described above), except for the increased OPF activity in the MT-null mice.

Regardless of its neural basis, the basis of neurobehavioral toxicity should be sought in early life stages when the brain Hg concentration is highly elevated (approximately 1.5 μg/g at its peak). Some *in vivo* experiments have demonstrated several candidate mechanisms of perinatal exposure to MeHg, including abnormal migration of neurons and/or glias (Kakita et al. 2003; Rodier et al. 1984), but at higher Hg concentrations. Using exactly the same exposure protocol as the present study, we found suppressed activity of type III iodo-thyronine deiodinase, a thyroid hormone-metabolizing enzyme, in the brains of PND10 mouse neonates (Mori et al. 2006), consistent with our previous study of higher MeHg doses (Watanabe et al. 1999). This perturbation could be one of the candidate mechanisms responsible for the later anomalous behaviors because even a transient change in thyroid hormones during the critical period of perinatal life exerts long-term consequences (Auso et al. 2004).

The effects of MeHg at 52 weeks of age were influenced by two potential modifying factors, sex and strain. In the OPF, while the locomotion was affected in both strains (although the direction was opposite), center occupancy was significantly increased only in the MT-null mice. In addition, the effects on PA were significant only in MT-null mice, whereas MeHg at a higher dose was reported to worsen PA performance in rats (6–8 weeks of age; Sakamoto et al. 2002). In addition, body weight gain was suppressed in both male and female MT-null mice, whereas in wild-type mice the suppression was observed only in males. Taken together, the MT-null strain appeared to be slightly more susceptible to the late-emergent effects of MeHg. Several lines of evidence have shown that MT-I,II is protective against the toxicity of MeHg (Leiva-Presa et al. 2004; Yao et al. 2000), and the present results were basically consistent with these reports. We have also reported the susceptibility of the MT-null strain to the neurotoxic effects of metallic Hg (Yoshida et al. 2004, 2006).

The difference in the susceptibility to MeHg between sexes is still debated (Clarkson and Magos 2006; National Research Council 2000; Vahter 2007). In the present study, some responses to MeHg were different between the sexes, including OPF performance at 12 and 52 weeks of age and MM performance at 52 weeks. The fact that the MT-null female group was the only group affected by MeHg at 12 weeks may suggest the particular susceptibility of females in this strain. This point needs to be clarified in further experiments.

The question remains of why MT-null mice are susceptible to the delayed neurotoxicity of perinatal MeHg. Apparently kinetics play only a minor role because the strain did not show distinct effects on the brain Hg concentration at PND10. The significantly lower brain Hg concentration in MT-null mice compared with corresponding wild-type mice at 13 weeks of age indicated that MT-I,II might play a significant role in the retention of Hg (or MeHg). This is consistent with the results of studies of metallic Hg exposure (Yoshida et al. 2004, 2005); a lower brain Hg concentration may not guarantee lower toxicity, supporting the protective role of the protein. Earlier studies suggest that brain MT-I,II has an important role both in the response to oxidative injury (Potter et al. 2007) and in the process of aging (Kojima et al. 1999). Therefore, the lack of MT can exaggerate the toxicity of MeHg by enhancing the initial effects due to oxygen radicals and/or by accelerating functional aging. Apart from this, an intriguing possibility is that the brain-specific isoform, MT-III, might contribute to the results we obtained because the expression of MT-III, together with MT-I, is increased in the brain of old rats, resulting in the low availability of free zinc for synapses (Mocchegiani et al. 2004). The age-dependent expressions of MT isoforms might be modified in MT-null mice.

Results of the present study might allow the possibility of alternative interpretations due to some potentially confounding factors. For example, except for the wild-type females, we observed significant differences in body weight between MeHg-exposed and non-exposed groups. Because these differences only became clear later in life, they might be associated with the toxicity that also emerged later in life. Manipulation of body weight in rodents alters activity levels, although the reported results are not always consistent with each other (Harrison and Archer 1987; Samuelsson et al. 2008). Also, the differential performance in PA could be related with the potential effects of MeHg on (electric) shock sensitivity, which we did not examine. At least one high-dose study with adult rats showed reduced electric sensitivity due to mercury exposure (Wu et al. 1985). These possibilities need to be addressed in future studies.

To summarize, the present results suggest that an initial (or triggering) toxicologic event occurs before the brain Hg concentration stabilizes and that the nature of this event should be either an acceleration of the aging process or interaction with the aging process. Thus, by identifying the physiologic events associated with the functional aging of the examined behavioral tasks, the fundamental toxicologic scar might be revealed.

## Figures and Tables

**Table 1:** Brain Hg concentrations in offspring after perinatal exposure to MeHg.

	Males		Females	
	Wild type	MT-null	Wild type	MT-null
PND10				
MeHg	384 ± 176	486 ± 120	435 ± 61	623 ± 76
Control	2 ± 1	2 ± 1	2 ± 1	2 ± 1
PNM3				
MeHg	5 ± 1	4 ± 1	6 ± 1	3 ± 1
Control	6 ± 1	4 ± 1	7 ± 1	4 ± 1

PNM, postnatal month. Values shown are mean ± SD of total Hg concentration (ng/g tissue); *n* = 3–4 mice per group.
